# Facilitators and barriers in Academic-Practice Partnerships (APPs) between Approved Educational Institutions (AEIs) and Residential Aged Care Facilities (RACFs) during pre-registration nursing students’ placements: a systematic review protocol

**DOI:** 10.1186/s13643-025-02877-1

**Published:** 2025-07-05

**Authors:** Ashley Chivaura, Iseult Wilson, Deborah Rainey, Wai Yee Amy Wong

**Affiliations:** 1 School of Nursing and Midwifery, Queen’s University Belfast, 97 Lisburn Rd, Belfast, BT9 7BL UK; 2 Norwich Medical School, University of East Anglia, Norwich Research Park, Norwich, Norfolk NR4 7TJ UK

**Keywords:** Pre-registration nursing students, Practice placements, Academic Practice Partnerships, Residential aged care facilities, Collaboration, Partnership, Nursing education, Nursing homes, Gerontological nursing, Long term care, Care home

## Abstract

**Background:**

Effective Academic-Practice Partnerships (APPs) between Approved Educational Institutions (AEIs) and practice learning partners are essential for enhancing the integration of the theoretical and practical aspects of nursing education. Recent literature regarding nursing student placements in Residential Aged Care Facilities (RACFs) highlights the significance of these partnerships and a need to explore the dynamic partnerships between AEIs and RACFs to better understand their role in improving student experiences and learning outcomes. Despite the recognition of the significance of understanding these APPs, this remains a significant gap in the existing literature.

**Methods:**

We will conduct a systematic review to identify facilitators and barriers to maintaining productive APPs between AEI and RACFs during pre-registration nursing student placements. Our research will involve an electronic literature search within MEDLINE, CINAHL, Web of Science, PsycINFO, and ERIC. Two independent reviewers will screen eligible papers, and a third reviewer will resolve any conflicts based on predefined inclusion and exclusion criteria. Eligible studies must have been published in English after 1990, and they should specifically address collaborative efforts between AEIs and RACFs in the context of pre-registration nursing student placements. All studies will be appraised using Caldwell’s Framework. Findings will be extracted using a standardised extraction table, and an in-depth synthesis will be conducted using thematic analysis.

**Discussion:**

This systematic review addresses a gap in the literature by identifying key factors that influence APPs in the context of RACF placements. The findings from this review have the potential to guide improvements in pre-registration nursing education by informing, nurturing, and supporting collaborative relationships between AEIs and RACFs, which promote enriched experiences for nursing students during their RACF placements. The review will also provide both clinical and academic educators with a deeper understanding of what facilitates and hinders collaboration, which will empower them to facilitate effective collaboration and develop their shared capacity in supporting students. Strengthening the partnerships between AEIs and RACFs fosters a shared vision and enhances the quality of pre-registration nursing education, which improves the standard of care for RACF residents.

**Supplementary Information:**

The online version contains supplementary material available at 10.1186/s13643-025-02877-1.

## Background

Academic-Practice Partnerships (APPs) are generally defined as strategic inter-organisational relationships aiming to advance mutual interests in practice, education, innovation, and research [1, 2]. In the context of nursing education, APPs began to develop in the twentieth century when nursing education transitioned from hospital-based formation to academic institutes. It is argued that this separation has led to a profound chasm between the theoretical and applied practice of nursing [3], potentially causing a lack of shared vision and missed opportunities in cases where APPs lack structure or formality. When this happens, it is likely to impede student learning outcomes and the experience of pre-registration nursing placements [3, 4].

Recent literature reviews on Residential Aged Care Facilities (RACFs) such as long-term care facilities, nursing homes, care homes, and assisted living facilities, which offer skilled nursing practice placements, emphasize the importance of understanding the nature of these APPs and their impact on student placement outcomes [5–7]. APPs are recognised as useful tools to reduce discrepancies in the provision of care and subsequently the associated healthcare costs [8]. Formalising APPs can also facilitate the implementation of potential improvements identified through ongoing research [2, 6].

In the context of nursing education, APPs with RACFs present unique contextual challenges, such as the extent of collaboration between different RACFs and approved educational institutions (AEIs), and the impact of organisational culture on collaboration and learning within specific institutions [9, 10]. In comparison with hospital placements, RACFs often have fewer registered nurses and are increasingly relying on agency workers, which may result in additional pressures on staff nurses to supervise students or place more responsibilities on students to identify and benefit from the available learning opportunities [11, 12]. However, McCloskey et al. [5] argue that the challenges faced by staff nurses in RACFs could also be mitigated through greater collaboration with universities. Strong partnerships are crucial for AEIs and practice partners, such as RACFs, to consolidate resources and create a conducive learning environment that promotes optimal patient care, supports nurses, and provides students with positive clinical experiences [16].

In the United Kingdom (UK), nursing students are required to complete clinical placements in diverse healthcare settings to meet the Nursing and Midwifery Council (NMC) proficiency standards. This diverse exposure not only enriches their experiences but also contributes to nurturing a future nursing workforce with a deep understanding of the diverse needs of patients across different ages [14]. Placements within RACFs provide future nurses with the opportunity to develop a deeper understanding of the older adult patient population who have a higher prevalence of chronic illnesses, multi-morbidity, and decreased capacity for self-care [15–17]. However, older adult care settings are often shown to be the least preferred career choice among nurses and one of the least preferred placement choices for nursing students [7]. Nursing in RACFs is often associated with negative stereotypes, including being perceived as uninteresting, offering limited career progression, and having poor staffing levels [6]. Similarly, a significant proportion of students perceived RACF placements as unattractive, repetitive, unskilled, unchallenging, and boring [4, 18]. Consequently, students may feel that RACF placements limit their professional growth and put them at a disadvantage compared to their peers in fast-paced acute care environments [11].

The negative perception of aged care has been attributed to multiple factors, including a negative portrayal of RACF in the media and ongoing negative stereotypes perpetuated within the AEIs that predominantly focus on acute care in hospitals within the nursing education curriculum [19, 20]. In addition, there is a misperception that RACF placements are only suited for teaching students basic nursing care [6]. Husebø et al. [6] argued that significant learning, which nurtures the formation of desirable nursing knowledge and attitudes, is possible within RACFs. However, potential challenges, including poor coordination between some AEIs and RACFs, can hinder the full potential of RACF placements as effective learning environments for pre-registration nursing students [5, 6]. Suboptimal student experiences during some RACF placements can further perpetuate negative attitudes towards aged care [6]. This highlights the importance of fostering collaboration between AEIs and RACFs while also cultivating a more comprehensive understanding of the distinct roles of pre-registration students and nursing educators in recognising and utilising the teaching and learning opportunities during placements in RACFs. These efforts are ultimately instrumental in empowering students to achieve the proficiency standards established by the NMC [14].

While challenges may arise, it is essential to emphasize that students acquire valuable skills and develop their capacity for providing compassionate and person-centred care during RACF placements [20]. RACF placements can enable nursing students to establish a solid foundation that significantly influences their nursing careers and can nurture graduates who are better able to advocate for patient autonomy and dignity [6, 20]. There is a recognition that some RACF facilities provide good experiences to students which helps change negative attitudes [11]. Therefore, it is important to understand both the factors facilitating positive placements and the barriers to fostering collaboration among different stakeholders involved in ensuring a positive learning environment. There is a need to ensure that staff feel supported in their supervisory roles within RACFs which will help safeguard against a shortage of experienced and passionate nurses working in RACFs [13]. By reviewing the literature, it would become possible to identify measures which can positively influence RACF placements.

### Research aim

This systematic review aims to identify the facilitators and barriers in sustaining the APPs between AEIs and RACFs in the context of pre-registration nursing students’ placements.

## Methods

This systematic review is part of the lead author’s doctoral research project. It will be guided by the Joanna Briggs Institute (JBI) manual for evidence synthesis [21], and adhere to the updated PRISMA 2020 statement guidelines for reporting systematic reviews [22], following the PRISMA 2020 checklist for systematic reviews [23]

### Eligibility criteria

#### Inclusion criteria

- Studies present facilitators and barriers in relation to pre-registration education of nursing students in RACFs.
- Studies discuss collaborative efforts between AEIs and RACFs.
- Articles published in peer-reviewed journals including primary studies, review articles, discussion papers and opinion pieces.
- Publication date ≥ 1990.

#### Exclusion criteria

- Studies from countries without a clear, distinct clinical placement component for pre-registration nursing students from academic institutions.
- Studies not written in English.

We will include all types of peer-reviewed studies illustrating facilitators and barriers in sustaining the APPs between AEIs and RACFs in the context of pre-registration nursing students’ placements, provided that these studies were published from January 1990 onwards and met the inclusion criteria since nursing education was primarily based on an apprenticeship model prior to 1990 [24]. Only peer-reviewed full texts published in English will be included due to limited resources available for translation.

### Search strategy

The lead author will develop the search strategy with guidance from a subject librarian in Nursing and Midwifery. The Population, Phenomena of Interest, Context framework (PICo) will be used to structure the review question as recommended by the JBI manual for systematic reviews with qualitative evidence (Table 1) [22]. The electronic databases searched for this review will include MEDLINE, CINAHL, Web of Science, PsycINFO, and ERIC. We will also cross-check the reference lists of all included studies to manually search for additional studies.

**Table 1:** Population, phenomenon of interest, context PICo framework

PICo	Key terms	Include other terms related to:
Population	Pre-registration nursing students	Pre-registration nursing students in baccalaureate, diploma, and undergraduate programmes
	Educators in nursing education	Educators in both academic and clinical settings
Phenomenon of interest	Academic Practice Partnerships (APPs) in relation to pre-registration nursing placements	Collaboration between universities and RACFs during nursing student placements
Context	Residential aged care facilities (RACFs)	Long-term care facilities, nursing homes, care homes and assisted living facilities

A proposed search strategy is attached in Additional file 1.

### Study selection

The search results from all electronic databases will be uploaded and managed using Rayyan, which is an online collaboration platform for researchers to screen papers for literature reviews [25]. After all the databases have been searched, all the results will be uploaded into Endnote and duplicate results will be removed before uploading the deduplicated search results into Rayyan. Two reviewers will screen titles and abstracts of the search results independently, followed by full-text screening against the eligibility criteria with documentation of reasons for exclusion. In the event of any discrepancies or disagreements, these will be first resolved by discussion to reach a consensus. A third reviewer will be consulted if required.

### Data extraction

Data from the included studies will be extracted by the lead author and checked by one of the co-authors using a standardised data extraction form. In the event of any discrepancies or disagreements, these will be resolved by discussion to reach a consensus. This form is developed based on the aim of the systematic review with the following preliminary items:

- Study characteristics (e.g., study design, country, setting, publication date)
- Participants’ characteristics (e.g., number of participants: pre-registration nursing students, nursing educators/assessors/supervisors and their roles)
- The level of collaboration between AEIs and RACFs will be allocated a score using the 7-point scale participation ladder by the Think Local Act Personal (TLAP) partnership [26]. The participation ladder describes seven levels of participation: coercing, educating, informing, consulting, engaging, co-designing, and co-producing. Coercing and educating offer limited user involvement. Informing, consulting, and engaging involve shallow involvement, where professionals design services with the recipient’s best interest in mind. Co-designing and co-producing involve shifting power toward the people and building a reciprocal relationship [25]. The TLAP participation ladder definitions will be used to allocate a score for the AEI and RACF relationship presented in each study [26]. Two reviewers will independently assign participation ladder scores, and a third reviewer will help resolve any discrepancies through discussion.
- Facilitators
- Barriers
- Study strengths and weaknesses

The authors will pilot the standardised data extraction form with the above preliminary items on three sample papers included in this systematic review. This is to ensure that all the relevant data to address the aim of the systematic review are obtained and to make any necessary modifications to the standardised form.

### Data analysis

The reporting of the data will follow the guidance of the JBI manual for evidence synthesis [21]. A descriptive analysis of the included studies will be conducted along with a narrative account of the key findings to address the aim of this systematic review. All the included studies will be critically appraised using Caldwell’s critique framework [27].

We will then conduct a thematic analysis using Braun and Clarke’s six-step model [28]: the first step involves familiarising ourselves with the data through reading and taking notes. Then we will generate codes by labelling meaningful segments to capture the explicit and implicit meaning of the data. Followed by looking for patterns to organise the generated codes into themes. We will then review and refine the themes for clarity, followed by naming the themes and creating a clear narrative for each theme. Finally, we will present the findings in a structured report [28]. This six-step model will help identify the common themes which will facilitate the identification of facilitators and barriers to sustaining productive APPs between AEIs and RACFs in the context of pre-registration nursing students’ placements.

## Discussion

This systematic review is the first, to the best of our knowledge, to explore the facilitators and barriers to collaborative partnerships between AEIs and RACFs in the context of pre-registration nursing student placements. Recent literature reviews note that this is a substantial gap in the literature [5, 7, 11, 13, 20].

Nursing students are shown to have diverse experiences during their practice placements, and common themes include dissatisfaction with supervision and the amount of time they got to spend with registered nurses, not feeling well-oriented to their RACF placements and the care routines, with some students feeling that their experience was insufficient or their mentors were not aware of their scope of practice [6]. Mentors were reported to have a high workload; they did not feel that they were supported and felt they had insufficient time to focus on teaching. They also indicated that they were expected to fulfil mentorship roles without adequate orientation [5]. These challenges identified within the literature indicate a mismatch of expectations from different stakeholders. By identifying barriers to collaboration, it is possible to mitigate a lot of these challenges through identifying where different stakeholders need support in fulfilling their respective roles, as argued by Mcloskey et al. [5]. By identifying facilitators to productive collaboration, it will become possible to reflect on the keys to successful partnerships, as strong partnerships are shown to help organisations develop impactful resources, create an environment capable of delivering the best quality of patient care, facilitate positive experiences for nursing students during clinical placements, and facilitate professional growth among nursing staff [13].

The findings of this systematic review will empower educators to consider implementing strategies which take into consideration the evidence surrounding what facilitates and hinders collaboration among the stakeholders involved in facilitating pre-registration nursing education related to RACFs whilst considering curriculum development and delivery. Students with a good understanding of APPs could better navigate the cultural differences between academic institutions and RACFs to see the bigger picture of how each organisation has a role to play and how a shared vision could be built through collaboration.

In conclusion, this systematic review will contribute to an understanding of RACF placements for pre-registration nursing students. By identifying facilitators and barriers in APPs between AEIs and RACFs, this review will help highlight how stronger partnerships between AEIs and Practice Learning Partners could be achieved. Ultimately the goal is to enhance the experience of nursing students during their RACF placements empowering them to provide high quality care to RACF residents.

## Supplementary Information

Additional file 1: A proposed search strategy.

## Abbreviations

AEIs: Approved Educational Institutions
APPs: Academic-Practice Partnerships
CINAHL: Cumulative Index to Nursing and Allied Health Literature
ERIC: Education Resources Information Center
JBI: Joanna Briggs Institute
MEDLINE: Medical Literature Analysis and Retrieval System Online
NMC: Nursing and Midwifery Council
PsycINFO: Psychological Information Database
RACFs: Residential Aged Care Facilities
TLAP: Think Local Act Personal
UK: United Kingdom
