# Supplementary material for: Facilitators and barriers in Academic-Practice Partnerships (APPs) between Approved Educational Institutions (AEIs) and Residential Aged Care Facilities (RACFs) during pre-registration nursing students’ placements: a systematic review protocol
Source: Syst Rev. 2025 Jul 5;14:137. doi: 10.1186/s13643-025-02877-1 (PMC12229029; doi:10.1186/s13643-025-02877-1)
Supplement: Supplementary file 1 — Additional file 1: A proposed search strategy. [file 13643_2025_2877_MOESM1_ESM.docx]

**Search Strategy**

| CINAHL |  |
| --- | --- |
| [TI]  [AB] | "Baccalaureate" OR "Diploma" OR "Nursing students" OR "Pre-registration nursing students" OR "Student" OR "Student attitude" OR "Student nurs*" OR "Student perspective" OR "Student perception" OR "Student satisfaction" OR "Undergraduate nursing education" OR "Undergraduate nursing student"  "Faculty experience" OR "Faculty perception" OR "Faculty perspective" OR "Facult*" OR "Instructor" OR "Lecturer" OR "Link lecturer" OR "Pre-licence" OR "Staff experience" OR "Teach*" OR "Supervisor"  [AND]  "Academic-community collaborations" OR "Academic Practice collaboration" OR "Academic-Practice partnerships" OR "Academic-service integration" OR "Academic-service partnerships" OR "Clinical education model" OR "Clinical placement" OR "Clinical practice" OR "Clinical practicum" OR "Collabor*" OR "Collaborative Learning" OR "Collaborative partnerships" OR "Collaborative practice models" OR "Community-based participatory research" OR "Cooperation" OR "Interprofessional collaboration" OR "Interprofessional relations" OR "Joint practice" OR "Joint Learning" OR "Learning model" OR "Nursing education model" OR "Nursing units" OR "Practicum" OR "Service-learning partnerships"  [AND]  "Aged care" OR "Assisted living" OR "Care home" OR "Care of the older facility" OR "Elderly care" OR "Geriatric care" OR "Geriatric nursing" OR "Geriatric*" OR "Gerontology" OR "Home for the aged" OR "Homes for the aged" OR "Long-term care" OR "Nursing home*" OR "Older adults" OR "Older people" OR "Residential aged care facilities" OR "Residential care" OR "Residential facility” |
| Medline ,Psych Info |  |
| [SH]  [Keywords] | “Nursing, Student” OR “Students Pre-Nursing”  [OR]  "Baccalaureate" OR "Diploma" OR "Nursing students" OR "Pre-registration nursing students" OR "Student" OR "Student attitude" OR "Student nurs*" OR "Student perspective" OR "Student perception" OR "Student satisfaction" OR "Undergraduate nursing education" OR "Undergraduate nursing student" OR "Faculty experience" OR "Faculty perception" OR "Faculty perspective" OR "Facult*" OR "Instructor" OR "Lecturer" OR "Link lecturer" OR "Pre-licence" OR "Staff experience" OR "Teach*" OR "Supervisor"  [AND]  "Academic-community collaborations" OR "Academic Practice collaboration" OR "Academic-Practice partnerships" OR "Academic-service integration" OR "Academic-service partnerships" OR "Clinical education model" OR "Clinical placement" OR "Clinical practice" OR "Clinical practicum" OR "Collabor*" OR "Collaborative Learning" OR "Collaborative partnerships" OR "Collaborative practice models" OR "Community-based participatory research" OR "Cooperation" OR "Interprofessional collaboration" OR "Interprofessional relations" OR "Joint practice" OR "Joint Learning" OR "Learning model" OR "Nursing education model" OR "Nursing units" OR "Practicum" OR "Service-learning partnerships"  [AND]  "Aged care" OR "Assisted living" OR "Care home" OR "Care of the older facility" OR "Elderly care" OR "Geriatric care" OR "Geriatric nursing" OR "Geriatric*" OR "Gerontology" OR "Home for the aged" OR "Homes for the aged" OR "Long-term care" OR "Nursing home*" OR "Older adults" OR "Older people" OR "Residential aged care facilities" OR "Residential care" OR "Residential facility” |
| Eric |  |
| [TI]  [AB] | "Baccalaureate" OR "Diploma" OR "Nursing students" OR "Pre-registration nursing students" OR "Student" OR "Student attitude" OR "Student nurs*" OR "Student perspective" OR "Student perception" OR "Student satisfaction" OR "Undergraduate nursing education" OR "Undergraduate nursing student" OR "Faculty experience" OR "Faculty perception" OR "Faculty perspective" OR "Facult*" OR "Instructor" OR "Lecturer" OR "Link lecturer" OR "Pre-licence" OR "Staff experience" OR "Teach*" OR "Supervisor"  [AND]  "Academic-community collaborations" OR "Academic Practice collaboration" OR "Academic-Practice partnerships" OR "Academic-service integration" OR "Academic-service partnerships" OR "Clinical education model" OR "Clinical placement" OR "Clinical practice" OR "Clinical practicum" OR "Collabor*" OR "Collaborative Learning" OR "Collaborative partnerships" OR "Collaborative practice models" OR "Community-based participatory research" OR "Cooperation" OR "Interprofessional collaboration" OR "Interprofessional relations" OR "Joint practice" OR "Joint Learning" OR "Learning model" OR "Nursing education model" OR "Nursing units" OR "Practicum" OR "Service-learning partnerships"  [AND]  "Aged care" OR "Assisted living" OR "Care home" OR "Care of the older facility" OR "Elderly care" OR "Geriatric care" OR "Geriatric nursing" OR "Geriatric*" OR "Gerontology" OR "Home for the aged" OR "Homes for the aged" OR "Long-term care" OR "Nursing home*" OR "Older adults" OR "Older people" OR "Residential aged care facilities" OR "Residential care" OR "Residential facility” |
| Web Of Science |  |
| [TI]  [Topic] | "Baccalaureate" OR "Diploma" OR "Nursing students" OR "Pre-registration nursing students" OR "Student" OR "Student attitude" OR "Student nurs*" OR "Student perspective" OR "Student perception" OR "Student satisfaction" OR "Undergraduate nursing education" OR "Undergraduate nursing student" OR "Faculty experience" OR "Faculty perception" OR "Faculty perspective" OR "Facult*" OR "Instructor" OR "Lecturer" OR "Link lecturer" OR "Pre-licence" OR "Staff experience" OR "Teach*" OR "Supervisor"  [AND]  "Academic-community collaborations" OR "Academic Practice collaboration" OR "Academic-Practice partnerships" OR "Academic-service integration" OR "Academic-service partnerships" OR "Clinical education model" OR "Clinical placement" OR "Clinical practice" OR "Clinical practicum" OR "Collabor*" OR "Collaborative Learning" OR "Collaborative partnerships" OR "Collaborative practice models" OR "Community-based participatory research" OR "Cooperation" OR "Interprofessional collaboration" OR "Interprofessional relations" OR "Joint practice" OR "Joint Learning" OR "Learning model" OR "Nursing education model" OR "Nursing units" OR "Practicum" OR "Service-learning partnerships"  [AND]  "Aged care" OR "Assisted living" OR "Care home" OR "Care of the older facility" OR "Elderly care" OR "Geriatric care" OR "Geriatric nursing" OR "Geriatric*" OR "Gerontology" OR "Home for the aged" OR "Homes for the aged" OR "Long-term care" OR "Nursing home*" OR "Older adults" OR "Older people" OR "Residential aged care facilities" OR "Residential care" OR "Residential facility” |
